# *StSN2* enhances tuber formation in potato via upregulating of the ABA signaling pathway

**DOI:** 10.3389/fpls.2025.1566237

**Published:** 2025-05-12

**Authors:** Shifeng Liu, Yichen Wang, Luopin Li, Lang Yan, Xiyao Wang, Qiang Wang, Xianjun Lai

**Affiliations:** ^1^ Panxi Crops Research and Utilization Key Laboratory of Sichuan Province, Xichang University, Liangshan, China; ^2^ School of Foreign Languages and Cultures, Xichang University, Liangshan, China; ^3^ Potato Research and Development Center, College of Agronomy, Sichuan Agricultural University, Chengdu, China

**Keywords:** potato, *StSN2*, ABA signaling, tuber formation, CRISPR/Cas9

## Abstract

Potato is a globally significant food and economic crop, playing a crucial strategic role in ensuring global food security and promoting economic development. The Snakin/GASA (gibberellic acid-stimulated Arabidopsis) family, a group of plant antimicrobial peptides regulated by hormones, play key roles in plant growth and development through hormone signal transduction. Previous studies have shown that overexpression of StSN2 significantly increases the tuber numbers and the proportion of large tubers, suggesting that StSN2 is a critical regulator of tuber formation, although its precise mechanism remains unclear. In this study,researchers utilized CRISPR/Cas9 technology to regulate the expression level of StSN2 in potatoes, and delved into the function of StSN2 in potato tuber formation. The research results show that deletion of the StSN2 gene led to a delay of about 14 days in the formation of potato stolons, and a decrease in yield by 20-30%. Bioinformatics analysis of the StSN2 promoter identified multiple cis-regulatory elements, and exogenous ABA and GA treatments confirmed that StSN2 responds strongly to ABA induction. Further analysis of key gene expression and enzyme activities during tuber development demonstrated that StSN2 enhances the ABA signaling pathway by upregulating components such as StPYL1, StSnRK2.2/2.3/2.6, and StABI5, thereby promoting tuber formation. In conclusion, this study integrates genetic, molecular, and physiological approaches to elucidate the regulatory role of StSN2 in potato tuber formation. The findings enrich our understanding of the molecular mechanisms underlying tuber development and provide a theoretical foundation for improving potato yields and stability through molecular design.

## 1 Introduction

The potato (*Solanum tuberosum* L.), a dicotyledonous annual herb belonging to the genus *Solanum* of the family Solanaceae, originates from the Andes Mountains in South America (Di et al., 2024). Renowned for its strong adaptability, tolerance to poor soil conditions, high yield, and comprehensive nutritional value, the potato is widely cultivated and serves as a globally significant food and economic crop (Mu and Sun, 2017). The development of the potato industry plays a pivotal role in addressing global food crises and improving food security. The primary edible part of the potato is its tuber, which forms through a complex developmental process. This process begins with the formation of stolons, which then undergo subapical swelling to produce tubers (Hannapel et al., 2017). Typically, a greater number of stolons correlates with increased tuber formation. However, not all stolons develop into tubers. Genetic factors, environmental conditions, and cultivation practices influence the tuberization rate of stolons, which typically ranges from 50% to 70% (Kolomiets et al., 2001). Given the importance of tuberization in potato productivity, investigating the mechanisms and regulatory pathways of tuber formation is crucial. Such research is essential for advancing potato breeding, optimizing cultivation strategies, and supporting the sustainable development of the potato industry.

The development of potato tubers is a complex process that is orchestrated by both internal physiological factors, such as the balance of plant hormones and sugar metabolism, and external environmental factors, including photoperiod, temperature, and nitrogen nutrition (Ding et al., 2024). Significant progress has been made in understanding the mechanisms regulating potato tuber formation. Among these factors, plant hormones are pivotal players due to their intricate interactions with environmental signals. ABA is a critical regulatory factor in potato tuber formation. StABL1, as a central transcription factor for abscisic acid (ABA) signaling, overexpression of *StABL1* promotes flowering and tuber formation in potatoes (Jing et al., 2022). ABA acts as an antagonist to gibberellins (GAs). Exogenous application of GA3 inhibits potato tuber formation, leading to decreased tuber yield, whereas application of GA biosynthesis inhibitors (such as choline chloride, paclobutrazol, or pyrimidinol) promotes tuber formation (Roumeliotis et al., 2012; Chen et al., 2022). Brassinosteroids (BRs) are important hormones that influence plant growth and development. Studies have found that overexpression of *StBRI1*, the core receptor for BR, positively regulates potato tuber development by increasing tuber number, weight, and cell size (Deng et al., 2024). BIN2 serves as a negative regulator in the BR signaling pathway, and overexpression of *StBIN2* enhances potato tuber formation by promoting hormone signaling and sugar metabolism, thereby increasing yield (Liu et al., 2023a). The balance of hormones and their signal transduction plays a pivotal role in tuber formation. The *StSN2* gene, part of the Snakin/GASA family, plays diverse roles in plant growth and development (Fan et al., 2017). Research has shown that overexpression of *StSN2* can induce potato tuberization and increase yield (Wang et al., 2021). In our previous research, we found that StBIN2 interacts with StSN2, and *StSN2* can promote the expression of *StBIN2*. *StBIN2* has been proven to promote tuber formation. Therefore, we have reason to believe that *StSN2* can also promote tuber formation. Exploring the molecular mechanisms of *StSN2* in tuber formation can provide valuable insights and support for breeding high-yield potato varieties.

CRISPR/Cas9 technology, known for its simplicity and cost-effectiveness, is a powerful tool for genome editing and has been successfully applied in various plants, such as Arabidopsis, sorghum, tobacco, and rice (Chavez-Granados et al., 2022; Hussain et al., 2018). Given the challenges in traditional potato breeding, including the crop’s tetraploidy and clonal propagation, CRISPR/Cas9 technology offers a promising approach for accelerating the breeding process. ARGOS8 is a negative regulator of ethylene response, and CRISPR/Cas9 editing of the *ARGOS8* gene enhanced drought tolerance in maize (Shi et al., 2017). A study has shown that the knockout of negative regulators of crop yield, such as the *GN1a*, *DEP1*, or *GS3* genes, significantly enhances specific traits associated with rice yield (Li et al., 2016). Potato is the third-largest food crop in the world after rice and wheat (Hameed et al., 2018) and plays a crucial role in maintaining global food security. Potato virus Y is a key factor limiting potato production, and resistance to the virus has been enhanced through CRISPR/Cas9 targeting of *eIF4E* (Noureen et al., 2022). Cultivated potatoes are tetraploids, highly heterozygous, and clonally propagated. Thus, developing new varieties through traditional breeding methods is a long-term and arduous endeavor. The application of CRISPR/Cas9 technology, however, will significantly shorten the breeding cycle of potatoes, providing a powerful impetus for the development of the potato industry.

As a globally significant food and economic crop, the potato’s tuber growth and development mechanisms are essential to understand for improving yields and ensuring food security. In this study, we investigated the role of *StSN2* in tuber formation and its associated regulatory mechanisms. Using potato genetic transformation technology, we generated gene-edited lines of *StSN2* and analyzed their tuber formation phenotypes. Our findings reveal that *StSN2* regulates tuber formation by responding to exogenous ABA signals and enhancing ABA signal transduction within tubers. Specifically, *StSN2* modulates the expression and enzymatic activity of key components of the ABA signal transduction pathway, including *StPYL1*, *StPP2C*, and *StSnRK2s*, thereby promoting tuber formation. This study provides novel insights into the regulatory role of *StSN2* in potato tuber development, offering a theoretical fundation for improving potato yields and advancing molecular breeding strategies.

## 2 Materials and methods

### 2.1 Plant materials and growth conditions

The experimental plant material used in this study was the common tetraploid potato variety Desirée (wild type, WT), introduced to China from the Netherlands by the Chinese Ministry of Agriculture. This variety has strong stress resistance, high yield, and a wide planting range. Virus-free *in vitro* seedlings of Desirée were provided by Xichang University. Sterile potato seedlings were propagated using plant tissue culture techniques. The seedlings were grown in tissue culture flasks containing Murashige and Skoog (MS) medium under controlled conditions in a growth chamber at 20°C with a 16-hour light/8-hour dark photoperiod. After three weeks of cultivation, the seedlings were transplanted into a substrate (latitude 30°70’N, longitude103°65’E) composed of coconut husks. Plants were regularly watered and tubers were harvested 90–100 days after transplantation. Observations and sampling of seedlings were conducted throughout the growth period.

Tobacco (*Nicotiana benthamiana*) seeds were sown in a substrate and grown for one month under the same conditions: 20°C with a 16-hour light/8-hour dark photoperiod. These plants were subsequently used for luciferase reporter assays.

### 2.2 Construction and identification of gene editing materials

To construct the *StSN2* gene-editing materials, potential off-target targets of *StSN2* (Soltu.DM.01G050660.1) were identified using the Cas-OFFinder tool (http://www.rgenome.net/cas-offinder/ (accessed on 22 July 2024)) to screen the target sequence and fuse it with guide RNA (gRNA). The selected recombinant gRNA was cloned into the *pAtU6-26-sgRNA-35S-EFP-Cas9* vector. The recombinant vector was transformed into *Agrobacterium tumefaciens strain* GV3101 via the freeze-thaw method (Weigel and Glazebrook, 2006). Transgenic potato plants were generated by infecting stem segments of the Desirée variety with the transformed *Agrobacterium* culture, following the method described (Wang et al., 2011). Potential positive transformants were screened using 50 ng/mL kanamycin, leaf genomic DNA was extracted using the CTAB method, and candidate transformants were identified using PCR amplification. The primers used for vector construction and validation are listed in **Supplementary Table S1**.

### 2.3 Bioinformatics analysis

The full-length gene of *StSN2* and the *StSN2* promoter region were retrieved from the potato database (http://spud-db.uga.edu/ (accessed on 22 July 2024)). Homologous proteins of the Snakin/GASA family from A. thaliana were downloaded from the NCBI database (https://www.ncbi.nlm.nih.gov/ (accessed on 25 July 2024)). Amino acid sequence homology was assessed using DNAMAN 6.0 software, and phylogenetic analysis was conducted using the neighbor-joining method in MEGA 7.0 software with 2000 bootstrap replications. These analyses were performed to elucidate the evolutionary relationship of StSN2 with other Snakin/GASA proteins and provide insights into its potential functional roles.

### 2.4 Sampling and processing methods

Tissue expression analysis: roots, stems, leaves, flowers, stolons, and tubers from potato tissue culture seedlings were sampled for tissue expression analysis.

The growth period of the WT potato is typically 95 days. After planting the tissue-cultured seedlings, we define the day of planting as day 0, based on the growth and development cycle of this variety. The period from 1 to 14 days after planting is designated as the seedling stage, from 14 to 28 days as the tuber initiation stage, from 28 to 56 days as the tuber bulking stage, and from 56 to 84 days as the tuber maturity stage. Starting from day 14, we randomly sampled and photographed the potatoes every 2 weeks.

At the end of the potato growth cycle (when more than half of the plant leaves turned yellow), a 5-point sampling method was used to isolate five plots (30 cm × 30 cm) of uniform size for harvesting.

### 2.5 RNA extraction and cDNA synthesis

Total RNA was extracted from various potato tissues using the SteadyPure Universal RNA Extraction Kit (AG, Changsha, China). RNA integrity was assessed via 1% agarose gel electrophoresis, and the RNA concentration was quantified with a Nano Drop One spectrophotometer (Thermo Scientific, Waltham, MA, USA). For cDNA synthesis, 1 μg of total RNA was used as the template for reverse transcription using the Evo M-MLV Reverse Transcription Kit (AG, Changsha, China), following the manufacturer’s protocol. The resulting cDNA was used for downstream gene expression analysis.

### 2.6 Quantitative Real-Time PCR (qRT-PCR)

The transcription level of *StSN2* was quantified using qRT-PCR. Reactions were performed with the SYBR Green Premix Pro Taq HS qPCR Kit on a 7500 Real-Time PCR System (Roche, Rotkreuz, Switzerland). The relative transcription levels were calculated using the 2^−ΔΔCt^ method (Livak and Schaittgen, 2001), with the elongation factor 1α-like (EF1αL) gene serving as the internal reference. Primer sequences used for the qRT-PCR analysis are listed in **Supplementary Table S1**. All experiments were conducted in three technical replicates to ensure accuracy and reliability of the data.

### 2.7 Hormone treatment and luciferase complementation assay

Six-week-old tobacco plants were used to evaluate the effects of hormone treatment. The promoter sequence of *StSN2* was cloned into the *pGreenII 0800-LUC* vector, and the recombinant vector was introduced into the GV3101 strain. Tobacco leaves were infected with *Agrobacterium* containing the recombinant plasmid and incubated in the dark for 24 hours. After incubation, the right half of each leaf was sprayed uniformly with 100 µM ABA and 50 µM GA until fully saturated (Berrocal-Lobo et al., 2002), while the left half served as the control. The hormone-treated plants were then placed under light conditions in a growth chamber for an additional 48 hours. Luminescence was captured using a CCD camera (Viber Fusion FX, France), and luciferase activity was quantified with a Dual Luciferase Reporter Gene Assay Kit (Vazyme, Nanjing, China) (Wang et al., 2022). All experiments were performed with three technical replicates to ensure the reliability and reproducibility of the results.

### 2.8 Enzyme activity assay

To assess the enzyme activities of PP2C and SnRK2 in potatoes, a plant ELISA kit (Kexing, Shanghai, China) was utilized. Initially, 100 mg of potato tuber bud eye tissue was extracted and homogenized. Subsequently, reagent buffer and HRP-conjugated reagent were added to the samples, which were then incubated at 37°C for 60 minutes. After incubation, a chromogenic luminescent reagent was introduced. Finally, the enzyme activities were measured at a wavelength of 450 nm (Liu et al., 2023b). The 100 mg sample consistsed of tissues from three potato budding eyes and three biological replicates were performed for these experiments.

### 2.9 Data analysis

All experiments in this study were performed with three biological replicates, and the results are presented as the mean ± SD (*n*=3). Statistical significance was assessed using Student’s t-test. In the figures, lowercase letters indicate samples with significant differences (*P ≤* 0.05). Data processing and statistical analyses were conducted using SPSS 24.0 software, while graphs and visualizations were created with Origin 2021.

## 3 Result

### 3.1 Phylogenetic and structural analysis of *StSN2*

**Figure 1:**
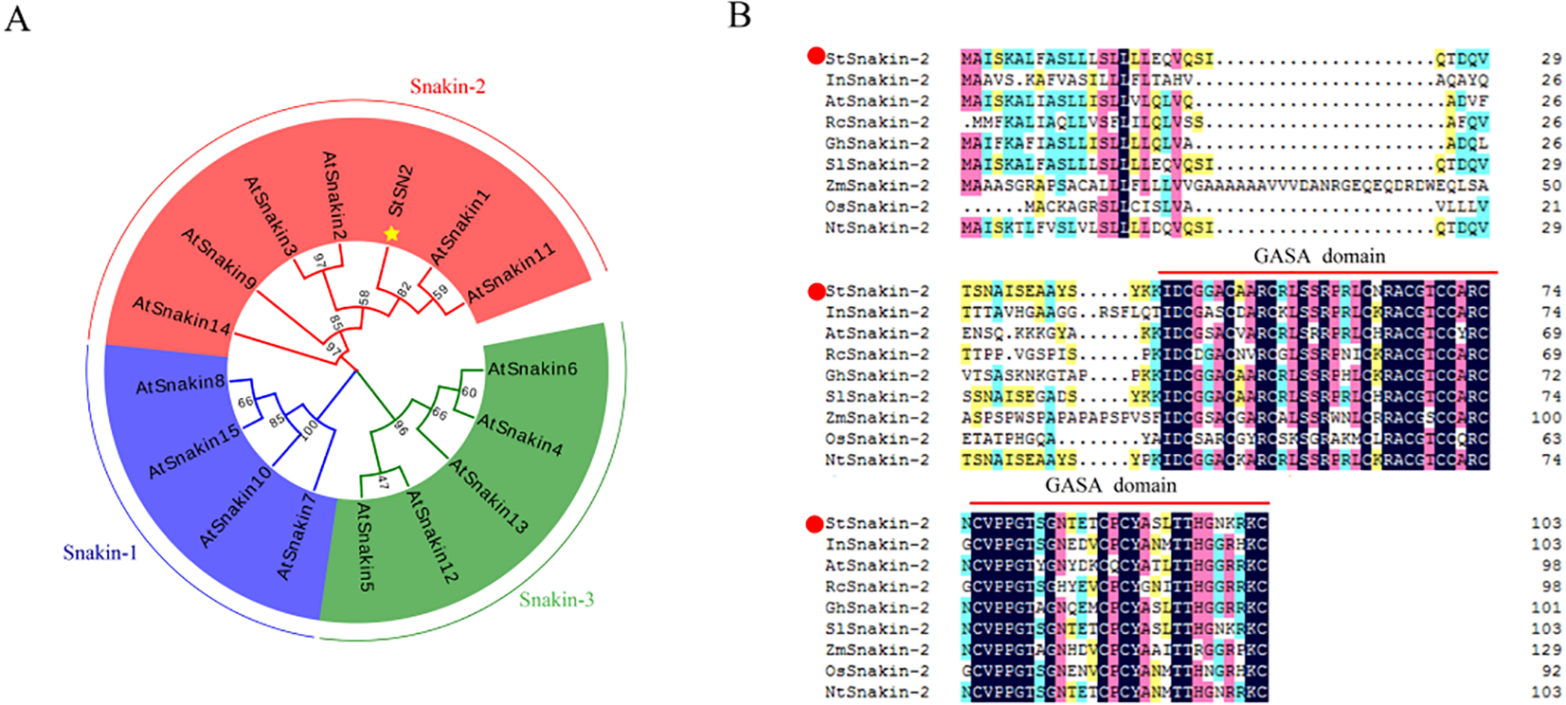
Evolutionary and structural analysis of potato StSN2 in comparison to the Arabidopsis GASA/Snakin family. **(A)** Phylogenetic tree analysis of the Arabidopsis GASA/Snakin family and potato StSN2. StSN2 is highlighted with a yellow star. Full-length amino acid sequences were downloaded from the **(A)** thaliana public TAIR database. **(B)** Comparative amino acids sequence analysis of Snakin-2 proteins across different species. StSN2 is marked with a red dot, and the GASA conserved domain is indicated by red solid lines.

Phylogenetic analysis was conducted to explore the evolutionary relationships between potato StSN2 and the Arabidopsis GASA/Snakin family. The analysis revealed that StSN2 was classified within Group II of the GASA/Snakin family (**Figure 1A**), consistent with previous research. This classification suggests that *StSN2* shares an evolutionary origin with other GASA/Snakin family members and may have conserved functions in plant growth and development. Further comparative sequence analysis showed that StSN2 contains a highly conserved GASA domain, a characteristic feature of the Snakin/GASA family. The alignment of Snakin-2 proteins from different species indicated that the GASA domain in StSN2 is conserved (**Figure 1B**). These findings reinforce the hypothesis that StSN2 has functional roles similar to other Snakin/GASA proteins and serves as a key regulatory element in potato growth and development.

### 3.2 Temporal and spatial expression analysis of *StSN2*

**Figure 2:**
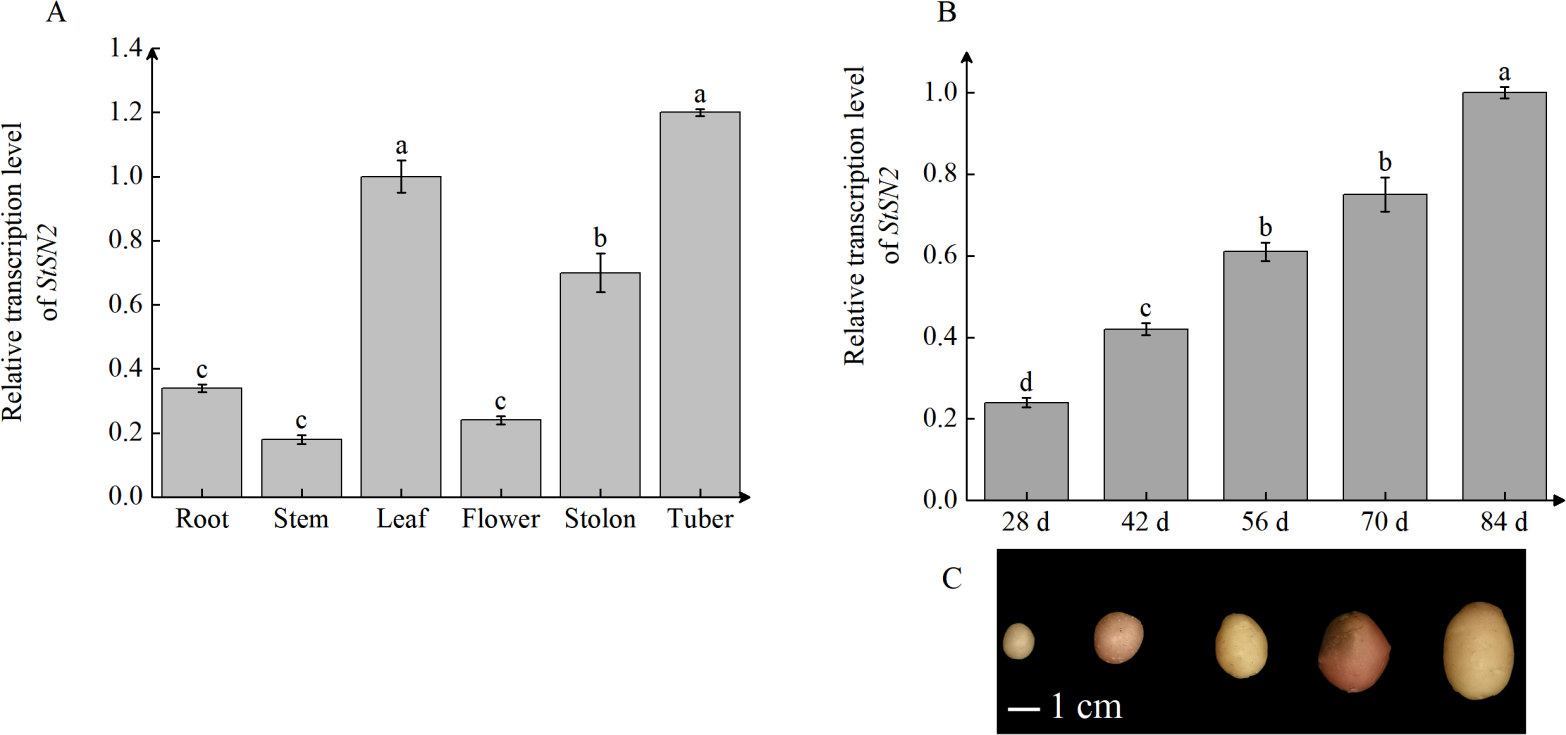
Tissue-specific and temporal expression patterns of *StSN2* in potato. **(A)** Transcription levels of *StSN2* in different potato tissues. Data were normalized using the 2^-ΔΔCt^ method and EF-1α was used as the internal reference for data normalization. **(B)** Transcription level of *StSN2* at different stages of tuber formation. **(C)** Representative images of potato tubers at various developmental stages. Data are shown as means ± SD (*n* = 3, Student’s t-test). Error bars represent the standard deviation of three replicates. Different lowercase letters indicate significant differences (*P* ≤ 0.05).

Tissue-specific expression analysis revealed that *StSN2* is expressed in all potato tissues, with the highest expression observed in leaves, followed by tubers and stolons, and relatively lower expression in roots and stems (**Figure 2A**). This broad expression pattern suggests that *StSN2* is involved in multiple physiological processes and may play a particularly significant role in the development of leaves, stolons, and young tubers. Temporal expression analysis during tuber formation showed that *StSN2* expression was low at the initial stage. However, its expression sharply increased after 40 days, coinciding with a critical period for tuber formation. By 60 days, the expression level reached its peak, 2.7 times higher than at the initial stage (**Figure 2B**). This timing corresponds with the development progression of potato tubers. Additionally, the degree of tuber development was consistent with the expression trends observed in qRT-PCR analysis (**Figure 2C**). In summary, these findings indicate that *StSN2* plays a vital role in tuber formation, with its expression closely linked to the critical stages of tuber development.

### 3.3 The absence of *StSN2* negatively impacts tuber formation

#### 3.3.1 Create CRISPR/Cas9-mediated StSN2 mutants.

**Figure 3:**
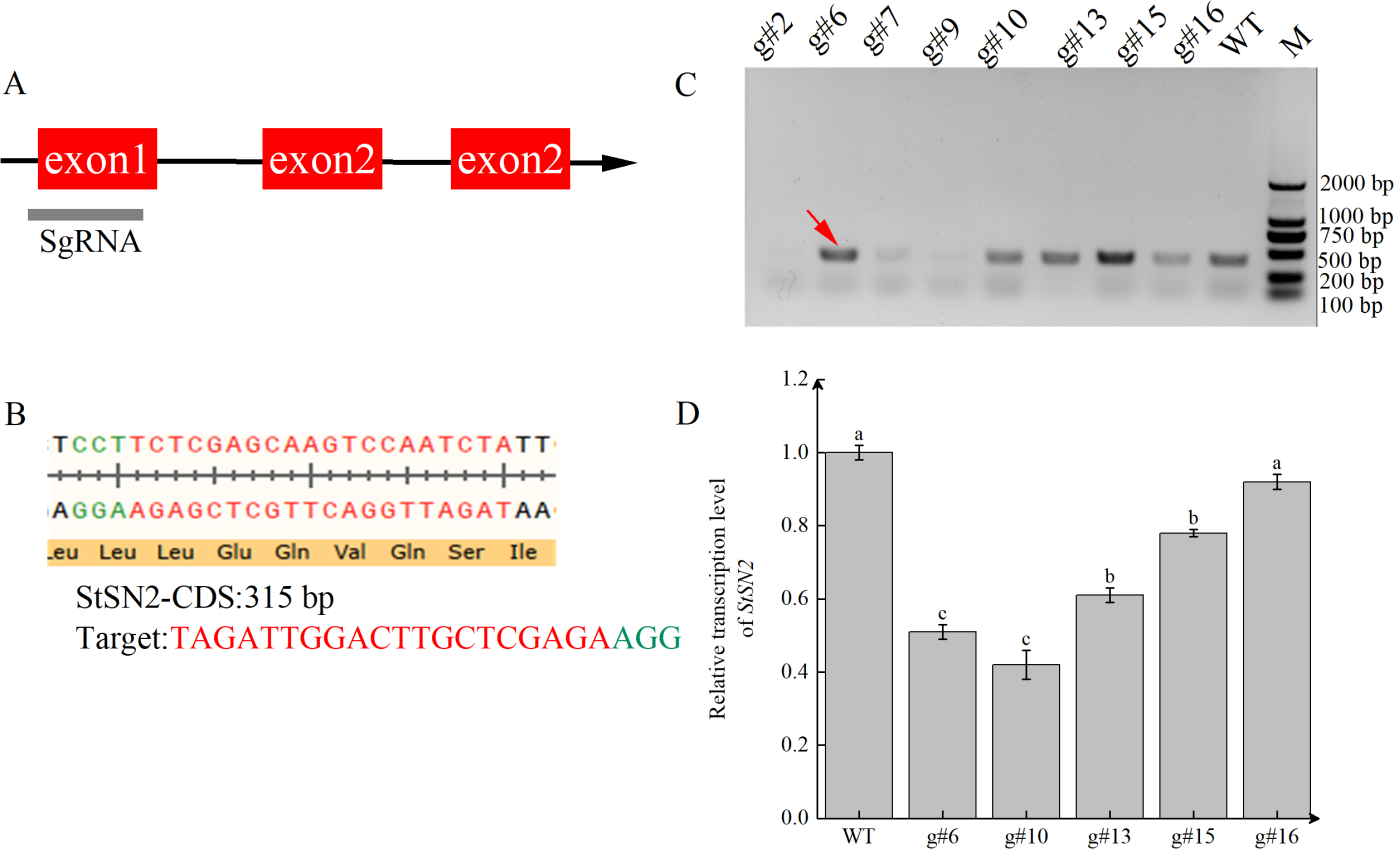
Generation and characterization of *StSN2* CRISPR/Cas9 mutants. **(A)** Schematic structure of *StSN2* showing the position of target sites designed for sgRNAs. **(B)** Sequence of the *StSN2* target region. **(C)** PCR identification of positive transformation material for *StSN2.* The red arrow indicates the *StSN2* sequence. **(D)** Transcriptional levels of *StSN2* in different mutant lines. Data were normalized using the 2^-ΔΔCt^ method, and EF-1α was used as the internal reference for data normalization. Error bars represent the standard deviation of three replicates. Different lowercase letters indicate significant differences (*P* ≤ 0.05).

To investigate whether *StSN2* is involved in tuber formation, we created a CRISPR/Cas9 gene-editing system targeting *StSN2*. Using Cas9 software, the target site was designed in the first exon region of the *StSN2* gene (**Figure 3A**), and the schematic representation of the target sequence editing is shown (**Figure 3B**). We performed PCR amplification on eight positive lines, and only five of these lines showed responsive bands (**Figure 3C**, as indicated by the red arrow). Subsequently, the transcription levels of *StSN2* were detected by qRT-PCR, revealing that the expression levels of *g#6* and *g#10* were the lowest (**Figure 3D**), although not completely absent. To investigate the mutations in *g#6* and *g#10*, PCR amplification and Sanger sequencing were performed on the target region of *StSN2*. After purifying the PCR products, they were ligated to the *pBM23* vector, and 18 clones from each line were sequenced. Mutations were successfully introduced into the target region through insertions/deletions (Indels) and single nucleotide polymorphisms (SNPs). In the mutated lines *g#6* and *g#10*, *StSN2* was targeted by gRNA, and the editing events were characterized by Sanger sequencing. The sequencing confirmed that in *g#6*, there were deletions of 1 bp, 4 bp, and 5 bp in the allele, and 1 bp was inserted; in *g#10*, there was a phenomenon of missing 3 bp and 5 bp, and inserting 1 bp (**Supplementary Figure 1**). Single nucleotide polymorphisms were observed in both *g#6* and *g#10*. In summary, we have successfully created mutant materials for *StSN2*, although they are not homozygous or pure materials.

#### 3.3.2 *StSN2* mutant shows inhibition of tuber formation

**Figure 4:**
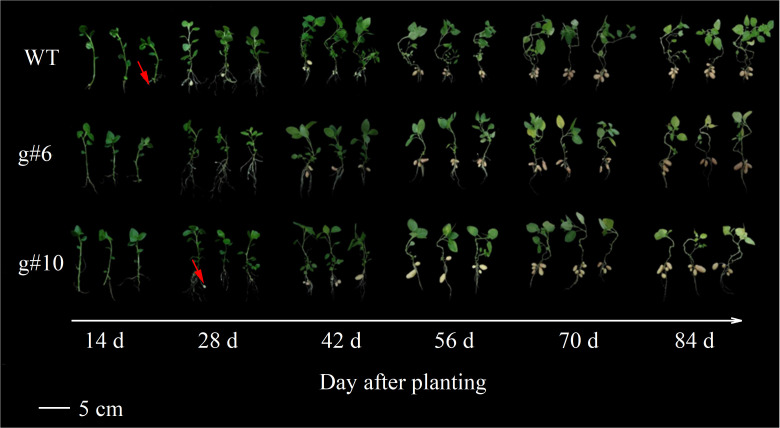
Effects of *StSN2* absence on phenotypic characteristics and tuber formation in potato the mutant lines. The red arrow points to the swollen stolon.

To investigate the impact of *StSN2* deletion on potato growth and tuber formation, the WT, *g#6*, and *g#10* lines were planted in a greenhouse under identical conditions. The development of subterranean stolons was observed and analyzed. The results showed that at 14 days, the stolons in WT plants exhibited significant swelling (as indicated by the red arrow). However, at this stage, neither *g#6* nor *g#10* showed any stolon formation. By 28 days, *g#10* began to exhibit swelling in its stolons (as indicated by the red arrow) (**Figure 4**). These observations suggest that the deletion of *StSN2* disrupts normal stolon development, delays tuber formation, and ultimately affects plant growth and yield.

**Figure 5:**
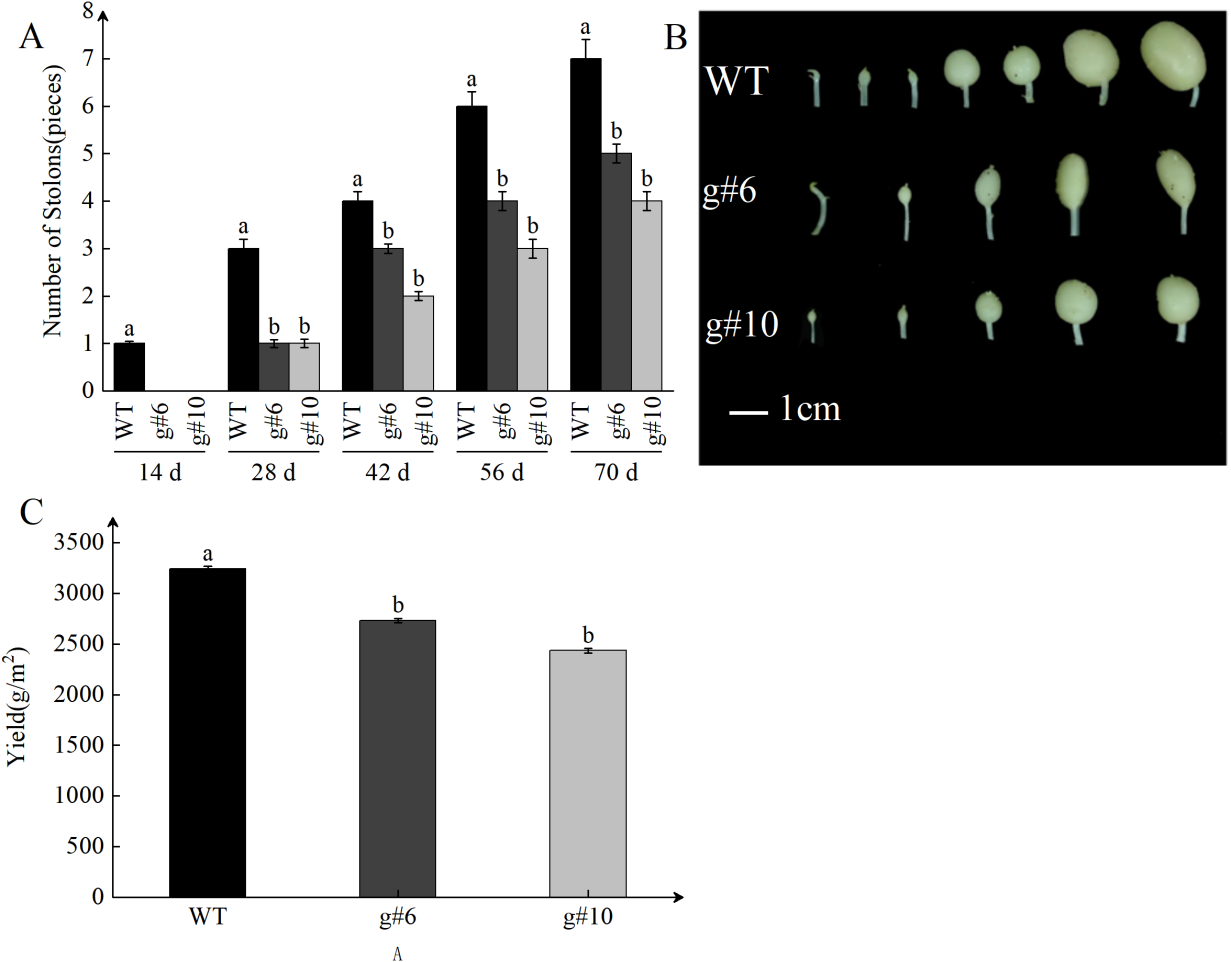
Impact of *StSN2* on stolon development and its role in promoting tuber formation. **(A)** Quantitative analysis of stolon numbers in *StSN2* mutant lines and WT. Error bars represent the standard deviation of three replicates. Different lowercase letters indicate significant differences (*P* ≤ 0.05). **(B)** Phenotypic comparison of stolons in *StSN2* mutant lines and WT plants at 70 days after planting. **(C)** Statistics of potato yield after harvest. Error bars represent the standard deviation of three replicates. Different lowercase letters indicate significant differences (*P*≤ 0.05).

The initiation and elongation of stolons are critical steps in potato tuber formation, significantly influencing tuber yield and quality. As plants grow, the number of stolons gradually increases, reflecting the plant’s vigor and the potential for higher tuber yield. Statistical analysis of stolon growth showed a significant upward trend in stolon number during development, consistent with the general growth pattern of potatoes (**Figure 5A**). At 70 days after planting, the number of stolons in the *StSN2* mutant lines (*g#6* and *g#10*) was significantly reduced by 25-40% compared to WT (**Figure 5B**), suggesting that *StSN2* regulates gene expression or metabolic pathways associated with stolon growth. Morphological observations further showed that stolon tips in the mutant lines exhibited poorer swelling compared to WT, emphasizing the critical role of *StSN2* in normal stolon development. After harvesting the potatoes, we conducted yield measurements and found that, compared to the WT, the yield of the *StSN2* mutant lines decreased significantly, being 0.7 to 0.8 times that of the WT (**Figure 5C**). Since stolon development directly impacts the final tuber yield and quality, these results strongly suggest that *StSN2* contributes to increasing stolon number and enhancing tip swelling, thereby promoting tuber formation and increasing yield.

### 3.4 Effects of *StSN2* on the formation of potato tubers

#### 3.4.1 ABA positively regulates the transcription of *StSN2*

**Figure 6:**
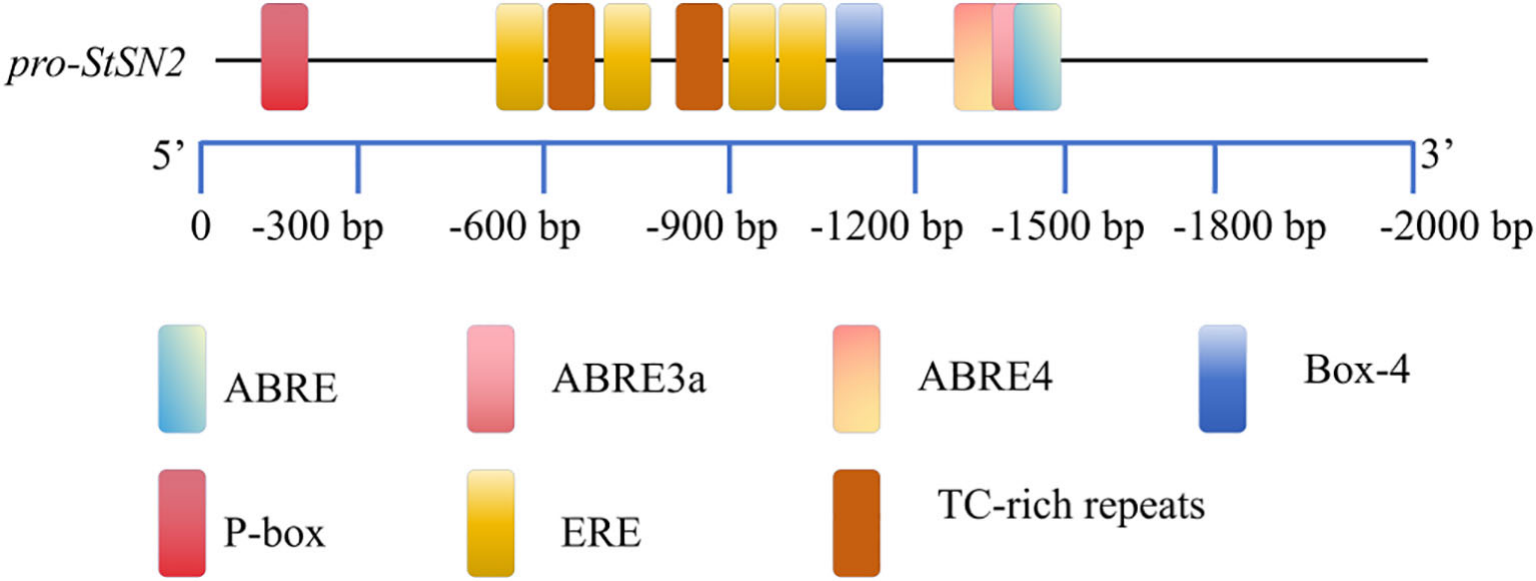
Identification and localization of CREs in the promoter region of *StSN2*. The names of the CREs are consistent with the names in **Table 1**. The boxes are labeled with the differential cis-acting elements and their locations.

To investigate the regulatory components of *StSN2*, we analyzed the 1800 bp upstream promoter region of the gene (**Figure 6**; **Table 1**). The results identified multiple hormone and stress-response cis-regulatory elements (CREs), including ABA-responsive elements such as ABRE, ABRE3a, and ABRE4, the gibberellin-responsive element P-box, and the stress-responsive element TC. These findings suggest that *StSN2* expression is regulated by ABA and GA signaling pathways, highlighting its potential role in hormonal responses during potato growth and development.

**Table 1:** Cis-regulatory elements of *proStSN2*.

Cis-element	Core sequence	Functional description of cis-element	Positions
ABRE	TACGTG	cis-acting element involved in abscisic acid responsiveness	-1439
ABRE3a	TACGTG	cis-acting element involved in abscisic acid responsiveness	-1439
ABRE4	CACGTA	cis-acting element involved in abscisic acid responsiveness	-1439
Box-4	ATTAAT	partof a conserved DNA module involved in light	-1237
ERE	ATTTTAAA	cis-acting elemen involved in ethylene responsiveness	-567, -1036, -748, -1094
TC-rich repeats	ATTCTCTAAC	cis-acting element involved in defense and stres	-799, -975
P-box	CCTTTTG	gibberellin-responsive element	-149

Other promoter cis-elements not related to this study are not listed in the **Table 1**.

**Figure 7:**
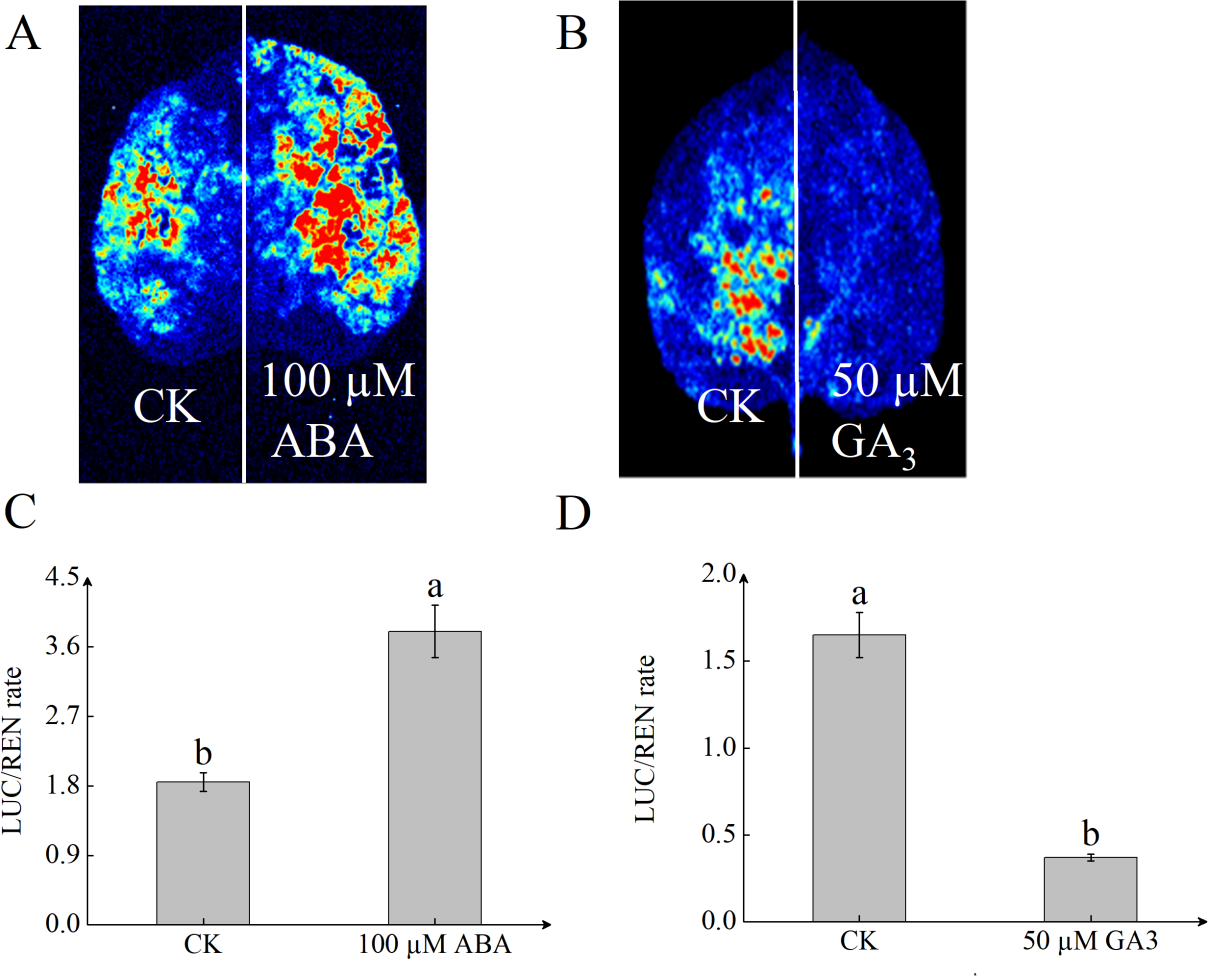
*StSN2* promoter activity is differentially regulated by ABA and GA3 treatments. **(A, B)** LUC immunostaining images of leaves from *pStSN2∷LUC* transient transgenic plants treated with 100 µM ABA **(A)** or 50 µM GA3 **(B)**. **(C, D)** Quantitative analysis of LUC activity in leaves treated with 100µM ABA **(C)** or 50µM GA3 **(D)**. Different lowercase letters indicate significant differences (*P ≤* 0.05).

To determine whether *StSN2* responds to hormone induction, we performed a luciferase (LUC) assay on the *StSN2* promoter. The results showed that ABA significantly increased the transcription activity of the *StSN2* promoter, with the LUC activity more than twice that of the control (CK). In contrast, GA inhibited the transcriptional activity of the *StSN2* promoter, resulting in significantly lower LUC activity compared to the CK (**Figure 7**). These findings indicate that *StSN2* is positively induced by ABA, while GA exerts a suppressive effect on its transcription.

#### 3.4.2 *StSN2* regulates tuber formation through the ABA signaling pathway

**Figure 8:**
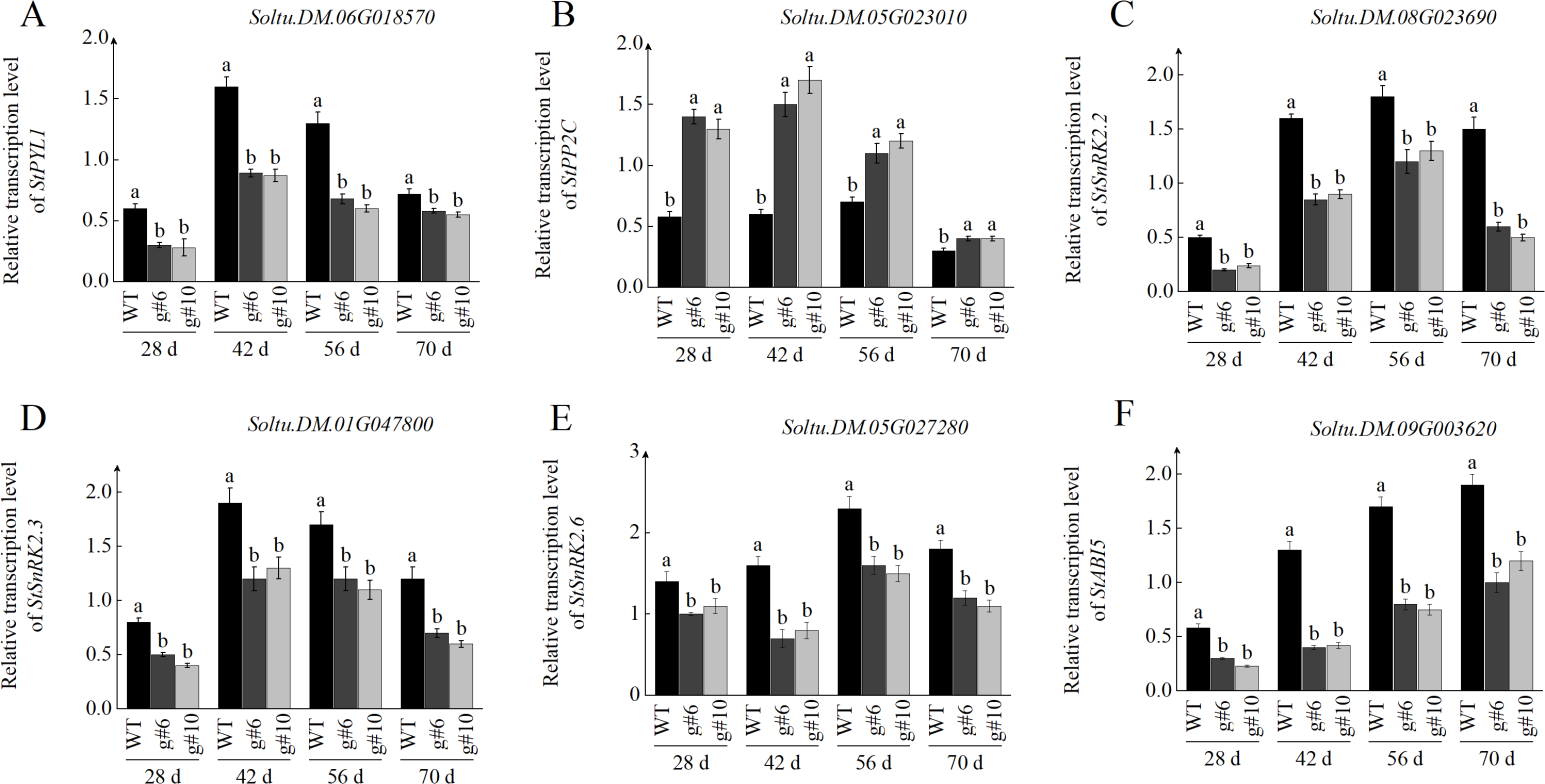
Effects of *StSN2* on transcriptional levels in the ABA signaling pathway during tuber formation. **(A–F)** Transcriptional levels of *StPYL1* **(A)**, *StPP2C* **(B)**, *StSnRK2* **(C)**, *StSnRK2.3* **(D)**, *StSnRK2.6* **(E)**, and *StABI5* **(F)**. Different lowercase letters indicate significant differences (*P* ≤ 0.05).

To investigate whether *StSN2* regulates tuber formation through the ABA signaling pathway, we measured the transcription levels and enzyme activities of key genes involved in ABA signal transduction during tuber formation. The transcription of *StPYL1*, a key ABA receptor, showed a similar trend in both WT and *StSN2* mutant lines, with levels increasing and peaking at 42 days before declining. However, throughout the growth period, the transcription level of *StPYL1* in *StSN2* mutants was significantly lower than in WT plants. These findings suggest that *StSN2* positively regulates ABA signal transduction, which is critical for tuber formation (**Figure 8A**). The transcription levels of *PP2C*, a negative regulator of the ABA signaling pathway, were significantly higher in *StSN2* mutant lines compared to WT during tuber formation. As tubers developed (from 28 to 70 days), the transcription of *StPP2C* gradually decreased, but the *StSN2* mutants consistently exhibited higher transcription levels compared to WT plants (**Figure 8B**). SnRK2 is a key component of the ABA signaling pathway and its transcriptional dynamics during tuber formation reveal spatio-temporal specificity. The transcription levels of *StSnRK2.2*, *StSnRK2.3*, and *StSnRK2.6* showed an initial increase followed by a rapid decline during the tuber formation. The transcription levels of *StSnRK2.2* and *StSnRK2.3* peaked around 42 days, whereas *StSnRK2.6* reached its peak later, suggesting distinct roles at different stages of tuber formation. Specifically, *StSnRK2.2* and *StSnRK2.3* are likely involved in early tuber formation (28–42 days), while *StSnRK2.6* plays a role in later stages such as tuber maturation and storage compound accumulation, which could reflect an intricate coordination of ABA signaling during early and late developmental stages (**Figures 8C–E**). Additionally, analysis of downstream ABA-responsive genes revealed that *StABI5* transcription gradually increased during tuber development in both WT and *StSN2* mutant lines. However, *StABI5* expression was significantly lower in *StSN2* mutants compared to WT, indicating that *StSN2* promotes ABA signal transduction by enhancing the transcription of *StABI5* (**Figure 8F**).In summary, *StSN2* positively regulates ABA signal transduction by modulating the transcription and activity of key ABA pathway components, ultimately promoting tuber formation and development.

**Figure 9:**
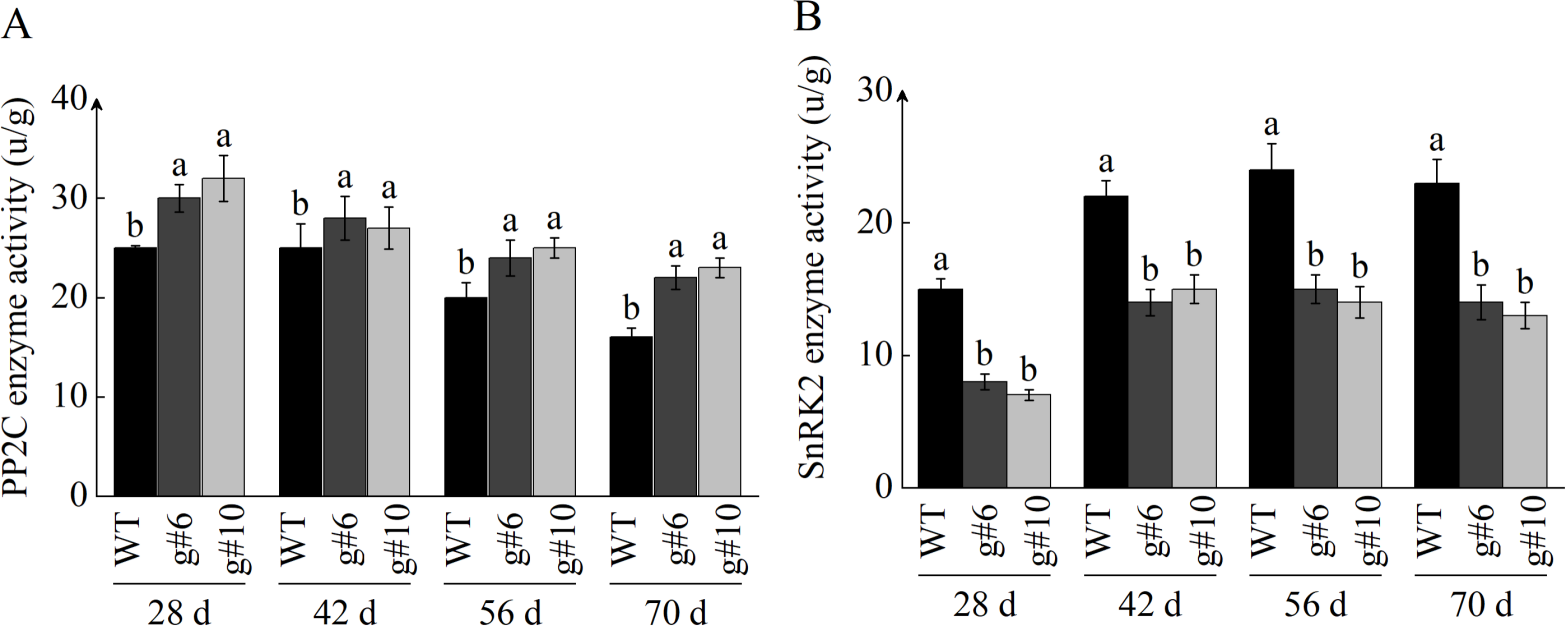
Effects of StSN2 on enzyme activities of StPP2C and SnRK2 during tuber formation. **(A)** Enzyme activity of PP2C protein. **(B)** Enzyme activity of SnRK2 protein. Different lowercase letters indicate significant differences (*P* ≤ 0.05).

In addition to examining the transcription levels of *PP2C* and *SnRK2*, we also assayed their enzymatic activities. The measurement results of PP2C phosphatase activity were consistent with those from qRT-PCR, revealing that the absence of *StSN2* led to an increase in PP2C phosphatase activity, which negatively regulates ABA signal transduction and inhibits tuber formation (**Figures 9A, B**). In contrast to PP2C, the enzymatic activity of SnRK2 was promoted by *StSN2*, positively regulating the ABA signaling pathway.

## 4 Discussion

The formation of potato tubers is a highly complex and finely regulated developmental process involving numerous physiological and biochemical activities, such as cell division, signal transduction, energy metabolism, and redox reactions (Ding et al., 2024; Abelenda et al., 2011). *StSN2*, a member of the GASA gene family, has been shown to play an important role in plant growth and development (Aubert et al., 1998; Berrocal-Lobo et al., 2002). In this study, we found that StSN2, like most Snakin proteins, contains a conserved GASA domain, indicating that *StSN2* may have similar functions to the *Snakin* gene family. The expression level of the gene in different tissues reflects its functional characteristics within the organism. *StSN2* exhibits the highest expression level in tubers, and its transcription level continuously increases as the potato grows through different developmental stages, indicating that *StSN2* plays a crucial role in tuber formation.

CRISPR/Cas9 has been applied as an efficient targeted mutation method in several different crops (Feng et al., 2013; Jiang et al., 2013). Here, we demonstrate that this method can be successfully applied to potato and achieve mutations in relevant genes. Prior studies have found that single-base insertions in the CRISPR/Cas9 system constitute the majority of events (Zhang et al., 2014). However, our results indicate that deletion events are more frequent than insertion events, and the *StSN2* mutant lines we created are heterozygous. These inconsistencies may be attributed to differences in Cas9 protein activity or expression levels, sgRNA expression levels, and/or the base composition of the target sequence. Therefore, in subsequent studies, we need to optimize the Cas9 protein and sgRNA sequences used in our experiments to generate better mutant materials.

*StSN2* is a crucial gene in potatoes, and our previous research has shown that this gene can maintain tuber dormancy through multiple pathways (Deng et al., 2021; Li et al., 2022; Liu et al., 2023b). In this study, we found that, compared to the control, the time for stolon swelling was extended by 14 days after the *StSN2* gene was mutated, and the number of stolons decreased by 25-40%, and the yield decreased by 20-30%. In summary, *StSN2* is essential for potato tuber and yield formation.

The regulation of gene expression is governed by upstream transcription factors, and the analysis of cis-acting elements provides crucial insights into the complexity and precision of gene expression regulation. Studies have shown that most *GASA* genes are induced by hormones. When potato stem segments were immersed in GA3 and ABA, it was found that under ABA treatment, the expression of *StSN2* increased with the duration of treatment, whereas under GA treatment, the expression level of *StSN2* decreased with time (Berrocal-Lobo et al., 2002). This indicates that *StSN2* responds to hormone induction. In this study, we discovered that the upstream promoter region of *StSN2* contains ABA and GA response elements. Luciferase assays further confirmed that *StSN2* is induced by ABA and inhibited by GA. These findings are consistent with earlier research, suggesting that *StSN2* may regulate tuber formation through the ABA pathway.

During the growth and development of potatoes, ABA plays a pivotal role, with its signaling pathway core consisting of the intracellular receptor, pyrabactin resistance-like proteins (PYLs), the co-receptor, clade A protein phosphatases of type 2Cs (PP2Cs), and sucrose non-fermenting-1 (SNF1)-related protein kinase 2s (SnRK2s) (Chen et al., 2020). When ABA binds to the receptor protein PYR/PYL, it triggers a series of reactions, most importantly lifting the inhibition of phosphatase PP2C on the activity of kinase SnRK2, thereby activating SnRK2 and downstream signal transduction processes (Chen et al., 2020). Recent research has found that overexpression of *StSN2* has a significant impact on ABA signal transduction (Liu et al., 2023b). Building on this, we further delved into the specific molecular mechanisms of *StSN2* in ABA signaling. The results showed that *StSN2* mutants exhibited a marked decrease in ABA signal transduction. Specifically, *StSN2* mutants significantly reduced the expression level of *StPYL1*, which, as a crucial component of the ABA receptor, plays a vital role in ABA signal transduction (Pizzio et al., 2022). The impairment of ABA perception and signal transduction further hindered the development of potato tubers. PP2C acts as a negative regulator in the ABA signaling pathway (Ma et al., 2009). In *StSN2* mutants, the expression of *StPP2C* was upregulated, and its enzymatic activity increased, further highlighting the disruption of ABA signal balance. Similarly, the transcription levels and activity of SnRK2s kinases, key components of ABA signal transduction, also showed a downward trend in the mutants, further confirming the role of *StSN2* as a positive regulator in the ABA signaling pathway. Plant hormones play a pivotal role in the formation of potato tubers, and besides them, several key genes also play important roles in the tuber formation process. *StSP6A* is a gene co-regulated by photoperiod and temperature, and its overexpression can effectively advance the occurrence of tuber formation (Bao et al., 2025). Similarly, *StBEL5* also plays a positive regulatory role in tuber formation (Kondhare et al., 2021). Specifically, after interacting with StPOTH1, StBEL5 jointly targets the promoter region of *GA2ox1* to inhibit its expression, thereby reducing the content of GA and promoting tuber formation (Kondhare et al., 2023). Furthermore, 14-3–3 proteins can form complexes with StSP6A proteins, and this interaction further participates in regulating the tuber formation process (Zhang et al., 2020). We examined the expression levels of genes such as *StSP6A*, *StGA2ox1*, *StBEL5*, *StPOTH1*, and *St14-3–3* at different stages of tuber development. The qPCR results were consistent with the tuber formation outcomes, showing that the expression levels of *StSP6A*, *StBEL5*, *StPOTH1*, and *St14-3–3* were significantly higher in WT plants compared to mutant materials, whereas *StGA2ox1* exhibited the opposite trend (**Supplementary Figure 2**). Thus, it can be inferred that the *StSN2* gene not only regulates tuber formation through the ABA signaling pathway but also further promotes tuber development by modulating the expression of genes related to tuber formation.

**Figure 10:**
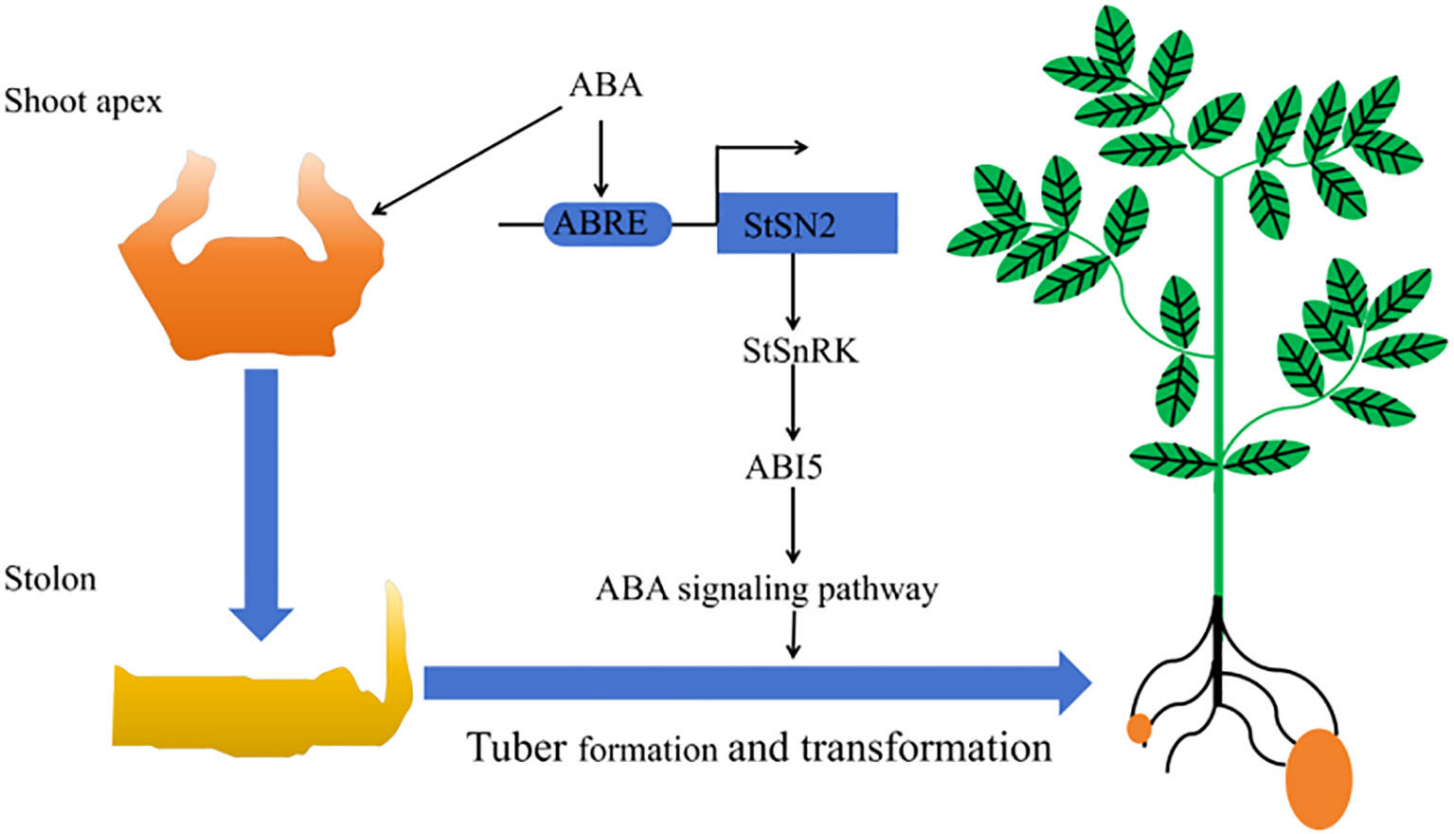
A working model illustrating how *StSN2* promotes tuber formation by enhancing the ABA signaling pathway.

In summary, CRISPR/Cas9-mediated *StSN2* editing provides valuable insights into the genetic regulation of potato tuber development. *StSN2* promotes tuber formation by modulating key components of the ABA signaling pathway, including *StPYL1*, *StPP2C*, *SnRK2s*, and *StABI5* (**Figure 10**). These findings provide a theoretical basis for molecular breeding to improve potato yield and quality. While this study primarily focused on tuber formation, further research is needed to determine whether *StSN2* directly interacts with other components of the ABA pathway or participates in cross-talk with additional signaling networks, such as ethylene or cytokinin pathways. Additionally, exploring *StSN2*’s role in plant growth, stress resistance, or tuber dormancy will be crucial to fully elucidate its agricultural potential and open new avenues for genetic improvement of potato yield and quality.

## Data Availability

The datasets presented in this study can be found in online repositories. The names of the repository/repositories and accession number(s) can be found below: https://www.ncbi.nlm.nih.gov/, AtSnakin15.
