# Supplementary material for: StSN2 enhances tuber formation in potato via upregulating of the ABA signaling pathway
Source: Front Plant Sci. 2025 May 12;16:1566237. doi: 10.3389/fpls.2025.1566237 (PMC12104193; doi:10.3389/fpls.2025.1566237)
Supplement: Supplementary file 1 [file Table1.pdf]

Potato *StSN2* promotes tuber formation by positively regulating the ABA pathway

Shifeng Liu, Luopin Li, Lang Yan, Qiang Wang, Xiyao Wang*and Xianjun Lai*

*** Correspondence:**
Corresponding Author: wangxy@sicau.edu.cn (X.W.); Tel.: +86-13980930138

Corresponding Author: 13547897810@163.com (X.L.); Tel.: +86-13547897810

Supplementary Data

S1. Quantitative RT-PCR for RNA sequencing validation.

Gene	Purpose	Forward/Reverse
StSN2	qPCR	TAACAGATGTAGCCACTGAC
		ACAACAAGTTCCACATGCCC
StSN2	CRISPR/Cas9	ATATATGGTCTCGTTTGTAGATTGGACTTGCTCGAGAAGGGTTTTAGAGCTAGAAATAGC
		CGATCTGGAAAATTTTGCAAAAAAAACGCGAACGCGTTGGAATTTGAAATATTTTTCT
Pro-StSN2	Luciferase	CTATAGGGCGAATTGGGTACCTGTAGTTGAACTTTTTATCA
		TAGAACTAGTGGATCCCCCGGGTGGAATTTGAAATATTTTTC
StSnRK2.2	qPCR	TTATGGAGTACGCAGCAGGT
		CCCACAGTCGACTTTGGTTG
StSnRK2.3	qPCR	TGTATGTCATGCTGGTGGGT
		ACGTTGATCTGGCTCCTCAA
StPYL1	qPCR	CGGTACCGGAAAACCCAGT
		CAATCTATGTTCACCGCCAAT
StSnRK2.6	qPCR	GATCGCATCTGTCAAGCTGG
		ACTTGGGACGTGAATGCAAC
StPP2C	qPCR	GGGACGGAGCTAGAGTTCTC
		ACCTGCCCACATCACAAAAC

File1: Amino acid sequences of the Arabidopsis Snake in/GASA family and StSN2

>StSN2

MAISKALFASLLLSLLLLEQVQSIQTDQVTSNAISEAAYSYKKIDCGGACAARCRLSSRPRLCNRACGTCCARCNCVPPGTSGNTETCPCYASLTTHGNKRKCP

>AtSnakin15

MKMPVVVQFFIISLLLTSSFYVLSSADSSACGGKCSVRCSKADRTHEECLEDCDICCQKCNCVPSGTYGNKDECPCYRDMKNSKGGSKCP

>AtSnakin9

MKKMNVVAFVTLIISFLLLSQVLAELSSSSNNETSSVSQTNDENQTAAFKRTY
HHRPRINCGHACARRCSKTSRKKVCHRACGSCCAKCQCVPPGTSGNTASCPC
YASIRTHGNKLKCP
>AtSnakin6
MAKLITSFLLLTILFTFVCLTMSKEAEYHPESYGPGSLKSYQCGGQCTRRCSNT
KYHKPCMFFCQKCCAKCLCVPPGTYGNKQVCPCYNNWKTQQGGPKCP
>AtSnakin1
MAISKALIASLLISLLVLQLVQADVENSQKKNGYAKKIDCGSACVARCRLSRR
PRLCHRACGTCCYRCNCVPPGTYGNYDKCQCYASLTTHGGRRKCP>AtSnakin
7
MKIIVSILVLASLLLISSSLASATISDAFGSGAVAPAPQSKDGPALEKWCGQKCE
GRCKEAGMKDRCLKYCGICCKDCQCVPSGTYGNKHECACYRDKLSSKGTPK
CP
>AtSnakin11
MAVFRVLLASLLISLLVLDFVHADMVTSNDAPKIDCNSRCQERCSLSSRPNLC
HRACGTCCARCNCVAPGTSGNYDKCPCYGSLTTHGGRRKCP
>AtSnakin12
MMKLIVVFVISSLLFATQFSNGDELESQAQAPAIHKNGGEGSLKPEECPKACEY
RCSATS
HRKPCLFFCNKCCNKCLCVPSGTYGHKEECPCYNNWTTKEGGPKCP
>AtSnakin8
MKLVVVQFFIISLLLTSSFSVLSSADSSCGGKCNVRCSKAGQHEECLKYCNICC
QKCNCV
PSGTFGHKDECPCYRDMKNSKGGSKCP
>AtSnakin5
MANCIRRNALFFLTLLFLLSVSNLVQAARGGGKLKPQQCNSKCSFRCSATSHK
KPCMFFC
LKCCKKCLCVPPGTFGNKQTCPCYNNWKTKEGRPKCP
>AtSnakin13
MATKLSIIVFSIVVLHLLLSAHMHPIYLESPARQPQPPQSQPQLPHHNNSQYGTT
QGSLQ
PQECGPRCGDRCSNTQYKKPCLFFCNKCCNKCLCVPPGTYGNKQVCPCYNN
WKTKSGGPKCP
>AtSnakin3
MAIFRSTLVLLLILFCLTTFELHVHAAEDSQVGEGVVKIDCGGRCKGRCSKSSR
PNLCLR ACNSCCYRCNCVPPGTAGNHHLCPCYASITTRGGRLKCP
>AtSnakin2
MAVFRSTLVLLLIIVCLTTYELHVHAADGAKVGEGVVKIDCGGRCKDRCSKSS
RTKLCLR
ACNSCCSRCNCVPPGTSGNTHLCPCYASITTHGGRLKCP
>AtSnakin14

MALSLLSVFIFFHVFTNVVFAASNEESNALVSLPTPTLPSPSPATKPPSPALKPPT
PSYK
PPTLPTTPIKPPTTKPPVKPPTIPVTPVKPPVSTPPIKLPPVQPPTYKPPTPTVKPPS
VQ
PPTYKPPTPTVKPPTTSPVKPPTTPPVQSPPVQPPTYKPPTSPVKPPTTTPPVKPP
TTTP
PVQPPTYNPPTTPVKPPTAPPVKPPTPPPVRTRIDCVPLCGTRCGQHSRKNVCM
RACVTC
CYRCKCVPPGTYGNKEKCGSCYANMKTRGGKSKCP
>AtSnakin4
MAKSYGAIFLLTLIVLFMLQTMVMASSGSNVKWSQKRYGPGSLKRTQCPSEC
DRRCKKTQYHKACITFCNKCCRKCLCVPPGYYGNKQVCSCYNNWKTQEGGP
KCP
>AtSnakin10
MKFPAVKVLIISLLITSSLFILSTADSSPCGGKCNVRCSKAGRQDRCLKYCNICC
EKCNY
CVPSGTYGNKDECPCYRDMKNSKGTSKCP

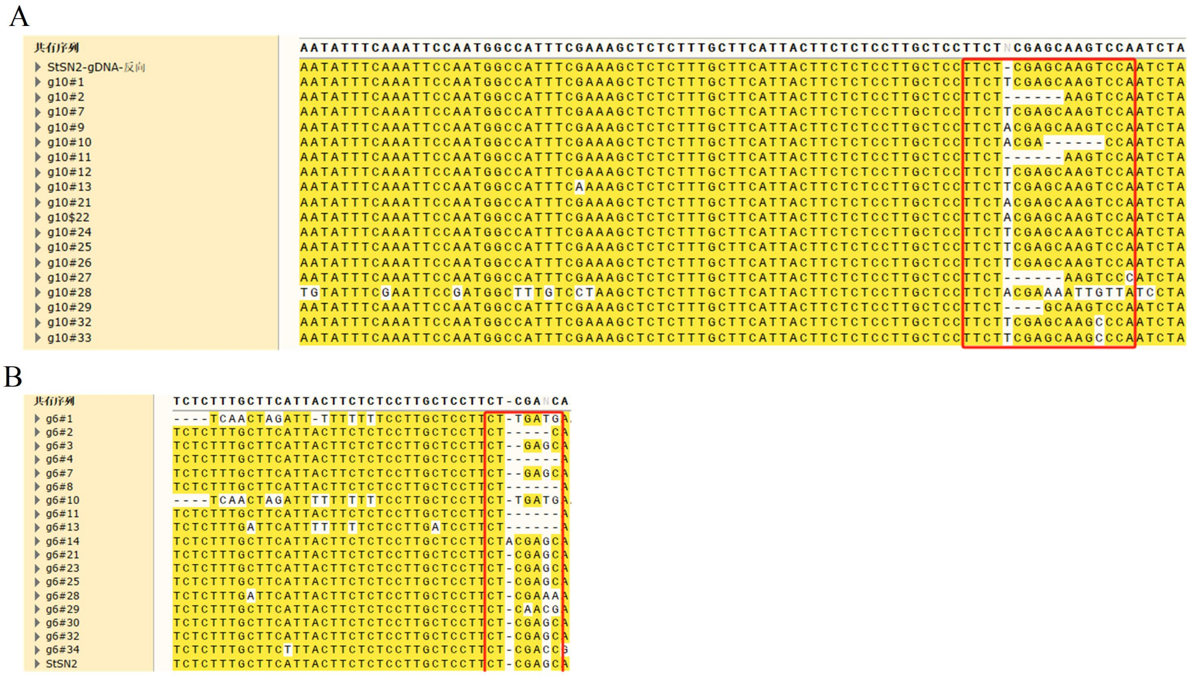

Supplementary figure 1. Sequence alignment of *StSN2* mutant strains.(A) The PCR identification result of *g#10*. The red box represents the target location. (B) The PCR identification result of *g#6*. The red box represents the target location.

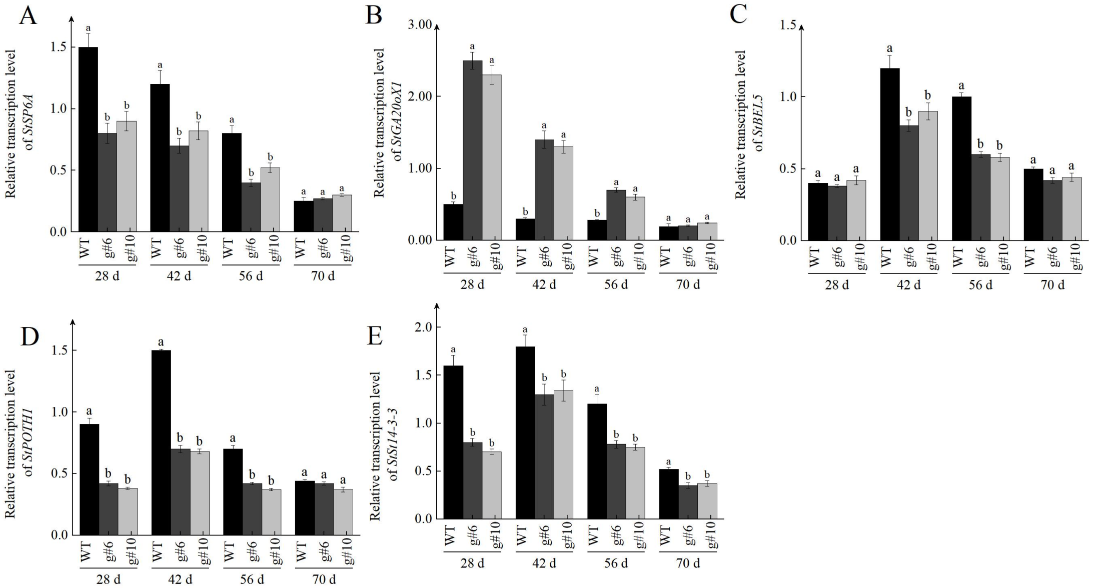

Figure 2. The effect of StSN2 on the transcription levels of related genes during tuber formation. (A-F) Transcriptional levels of *StSP6A* (A), *StGA20ox1* (B), *StBEL5* (C), *StPOTH1* (D), and *St14-3-3* (E). Different lowercase letters indicate significant differences ($P \leq 0.05$).
